# Induced Bacterial Cross-Resistance toward Host Antimicrobial Peptides: A Worrying Phenomenon

**DOI:** 10.3389/fmicb.2016.00381

**Published:** 2016-03-24

**Authors:** Osmel Fleitas, Octávio L. Franco

**Affiliations:** ^1^Programa de Pós-Graduação em Patologia Molecular, Faculdade de Medicina, Universidade de BrasíliaBrasília, Brazil; ^2^Programa de Pós-Graduação em Ciências Genômicas e Biotecnologia, Centro de Análises Proteômicas e Bioquímicas, Universidade Católica de BrasíliaBrasília, Brazil; ^3^Programa de Pós-Graduação em Biotecnologia, S-Inova Biotech,Universidade Católica Dom BoscoCampo Grande, Brazil

**Keywords:** antimicrobial peptides, cross-resistance, bacterial infection, antibiotics

## Abstract

Bacterial resistance to conventional antibiotics has reached alarming levels, threatening to return to the pre-antibiotic era. Therefore, the search for new antimicrobial compounds that overcome the resistance phenomenon has become a priority. Antimicrobial peptides (AMPs) appear as one of the most promising antibiotic medicines. However, in recent years several AMP-resistance mechanisms have been described. Moreover, the AMP-resistance phenomenon has become more complex due to its association with cross-resistance toward AMP effectors of the host innate immune system. In this context, the use of AMPs as a therapeutic option could be potentially hazardous, since bacteria could develop resistance toward our innate immune system. Here, we review the findings of major studies that deal with the AMP cross-resistance phenomenon.

## Introduction

With the discovery and introduction in the early 20th century of antimicrobial compounds for the treatment of infections caused by microorganisms it was thought that infections would no longer endanger human health. However, one century later, infectious diseases still constitute a threat (Saga and Yamaguchi, 2009).

Undoubtedly, the capacity of microorganisms to develop resistance to antimicrobial compounds has been one major cause of this situation. Therefore, the scientific community is deeply involved in the search for new and more powerful antimicrobial compounds that overcome pathogen resistance. In this search, among the most promising compounds that have been found are the antimicrobial peptides (AMPs). AMPs are short amphipathic peptides, generally cationic, produced by a wide variety of organisms that range from bacteria to humans. They perform antimicrobial activities by dissimilar mechanisms of action, including cell membrane permeability and inhibition of the synthesis of proteins, nucleic acids and the cell wall, among others (Jenssen et al., 2006; Guilhelmelli et al., 2013).

It is assumed that bacterial resistance to AMPs is unlikely because bacteria have to change conserved targets, such as the cell membrane, and this could be costly (Zasloff, 2002). However, the reality is quite different, since several AMP resistance mechanisms have been described (as reviewed in Maria-Neto et al., 2015). The resistance to AMPs would compromise their use and effectiveness as therapeutic agents. Moreover, within the issue of resistance to AMPs a more worrying concern has emerged, which is the potential that AMP therapy has to induce cross-resistance toward AMPs that are effectors of our innate immune system, and thus compromising our natural defense against pathogens. Bell and Gouyon (2003) debated the possibility that introducing AMPs as therapeutic agents may provoke the evolution of cross-resistance toward our own defenses; they called this “arming the enemy.” In this review, we will examine a range of studies focused primarily on cross-resistance toward AMPs of the innate immune system mediated by point mutations that arising during the exposition of bacteria to sub-lethal doses of therapeutic AMPs.

## Cross-Resistance Toward AMP Constituents of the Innate Immune System Induced by Therapeutic AMPs

In recent years, it has been posited that the exposure of bacteria to therapeutic AMPs can select AMP-resistant strains *in vivo* and *in vitro* and that the fitness cost associated with this resistance could be low, allowing their persistence (Saleh-Mghir et al., 2011; Dobson et al., 2013). A disturbing consequence of this is the fact that bacteria could cross-resist the microbicidal action of human AMPs on which the innate immune system depends. Although this last proposition has not been deeply explored, several studies that address this topic are starting to emerge.

Habets and Brockhurst (2012) showed that propagation of a nasal isolate of *Staphylococcus aureus* by serial transfer in medium supplemented with increasing concentrations of pexiganan (a synthetic AMP) allowed the emergence of resistant bacteria. Most of the evolved resistant bacteria increased minimal inhibitory concentrations (MICs) for pexiganan in comparison to the ancestral bacteria. Moreover, it was observed that resistance had an associated cost that translated into impaired growth rate and that could be compensated, allowing a resistant bacteria growth rate comparable with the ancestral bacteria and keeping the resistant status. Additionally, pexiganan-resistant bacteria cross-resist the action of human-neutrophil-defensin-1 (hNP-1). Interestingly, bacteria with moderate resistance to pexiganan and therefore a lower associated cost were the most cross-resistant to hNP-1, suggesting that cross-resistance could be selected if other mechanisms that are more effective in resisting the therapeutic AMP exist within the bacterial population.

**FIGURE 1:**
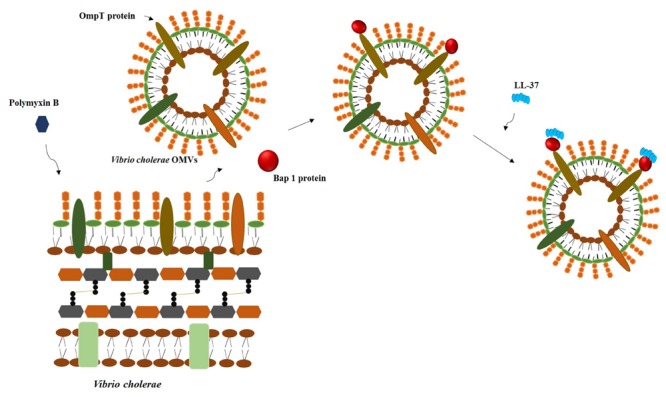
**The challenge of *Vibrio cholerae* with sub-lethal concentrations of polymyxin B induced the release of outer membrane vesicles that bind the protein Bap1, which in turn binds LL-37**.

Recently it was stated that heteroresistance could be involved in cross-resistance to the host innate immune system. Colistin-heteroresistant *Enterobacter cloacae* strains were cross-resistant to lysozyme after being treated with the bacteria-derived AMP colistin (Napier et al., 2014). Besides, Duperthuy et al. (2014) showed that the treatment of *Vibrio cholerae* culture with polymyxin B (a bacteria-derived AMP) induced the production and secretion of biofilm-associated extracellular matrix protein (Bap 1) which associated to outer membrane vesicles and in turn binds to LL-37 mediates cross-resistance toward this peptide (**Figure 1**). The secretion of outer membrane vesicles has been associated with resistance to AMPs and potentially could act at the population level (Manning and Kuehn, 2011). Thus, the findings of these studies suggest that population-based resistance mechanisms could be involved in the cross-resistance to AMPs of the innate immune system.

Other studies have also identified individual cells resistance mechanisms that could be involved in cross-resistance. A study by Napier et al. (2013) observed a high correlation between colistin resistance and cross-resistance to LL-37 and lysozyme in clinical isolates of *Acinetobacter baumannii*. Comparison between two pairs of *A. baumannii* clinical isolates from patients’ pre- and post-colistin treatment showed that pre-treatment isolates were sensitive to colistin, while the post-treatment isolates were resistant to this drug. Additionally, one of the post-treatment isolates presented cross-resistance to LL-37 and lysozyme while the other only to lysozyme. Non-synonymous mutations in the *pmrB* genes appear to be involved in the resistance and cross-resistance phenomenon. The gene *pmrB* codes for the protein PmrB, which together with PmrA protein integrates the two-component regulatory system PmrAB, forming part of the network that participates in the lipopolysaccharide (LPS) modifications (Chen and Groisman, 2013).

In another study on mutations, Lofton et al. (2013) showed that the serial passage in increased concentrations of LL-37 (human-derived peptide), CNY100HL (synthetic peptide) and wheat germ histones (mixture of different histones and shorter histone peptides) produced evolved resistance to these AMPs in *Salmonella typhimurium* LT2. Mutations in the *phoP* and *waaY* genes are involved in the cross-resistance to the three tested peptides. The *phoP* gene encodes the PhoP protein response regulator in the two-component regulatory system PhoPQ, and the *waaY* gene for WaaY kinase, which is responsible for heptose II phosphorylation in the LPS inner core. Additionally, it was observed that at low concentrations of the three AMPs the *waaY* mutant outcompeted the wild strain. This is of particular concern, because the concentrations required are within the range of AMP concentrations found in secretions near host epithelial cells. There have been recent tests investigating the effect of these mutations on the fitness of *Salmonella typhimurium* LT2 under several conditions *in vitro* that mimic the host environment and by growing inside a mouse host. The results suggested that the mutations had minor effects on the fitness and on the survival of mutants in the host (Lofton et al., 2015).

The above studies suggest that LPS modifications may be involved in the cross-resistance phenomenon. The LPSs are the major constituent of the surface of Gram-negative bacteria and are involved in the initial binding of cationic AMPs via electrostatic interactions. Therefore, changes in the charge state of LPSs could influence the initial binding of AMPs.

Recently, Bayer et al. (2015) studied the influence of single nucleotide polymorphisms (SNPs) within the multiple peptide resistance factor open reading frame (*mpr*F ORF) in resistance to the bacteria-derived AMP daptomycin (DAP) and cross-resistance to the host defense peptide thrombin-induced platelet microbicidal protein (tPMP). In the study, 22 daptomycin-susceptible (DAP-S) and daptomycin-resistant (DAP-R)^∗^ isogenic clinical methicillin-resistant *Staphylococcus aureus* (MRSA) strain-pairs were used. It was observed that most of the DAP-R strains had significantly higher survival faced with tPMP than DAP-S strains. Specifically, DAP-R strains that carried SPNs within the central bifunctional domain of MprF protein showed a higher survival when challenged with tPMP than DAP-R strains that carried SNPs at the C-terminal synthase domain of MprF. The MprF protein mediate the synthesis and translocation of lysyl-phosphatidylglycerol (positively charged phospholipid) from inner to outer leaflet of the cell membrane. Therefore, the presence of this positive-charged phospholipid in the outer leaflet of the cellular membrane could change the membrane charge, making it more positive and facilitating the repulsion of cationic AMPs (Ernst and Peschel, 2011).

Additionally, it was shown that in the DAP-R strains there was an increase in the synthesis of lysyl-phosphatidylglycerol and in the surface positive charge. However, no significant differences in the positive charge surface were observed between the strains with mutations in the central bifunctional domain of MprF and strains with mutations in the synthase domain. The same authors suggested that charge-mediated and unrelated mechanisms may be involved in cross-resistance. Interestingly, DAP-R strains that did not carry SPNs within *mprF* ORF were resistant to killing by tPMP, suggesting that mechanisms other than *mprF* ORF mutations could be involved in cross-resistance. Several studies have shown that DAP-R *S. aureus* strains are cross-resistant to host defense peptides like hNP-1 and LL-37 (Bayer et al., 2014; Mishra et al., 2014).

Methicillin-resistant *Staphylococcus aureus* bloodstream isolates from patients that had never been exposed to DAP treatment showed relatively high and medium DAP MIC values. The strains with higher DAP MIC showed increased resistance to killing by tPMPs but not to hNP-1. This suggested that exposure to certain host bloodstream factors, including host defense peptides, could select MRSA strains with high DAP MIC (Mishra et al., 2012). Recently, in a rabbit prosthetic joint infection model, it was observed that a MRSA isolate from DAP-naïve rabbit presented an increased MIC to DAP and significantly reduced killing by hNP-1 and tPMPs. As it is possible that the infecting DAP-S MRSA strain in this experimental model had been exposed to neutrophil-derived and platelet-derived AMPs, these results suggested that exposure to AMPs *in vivo* could select strains that are cross-resistant to DAP prior to DAP exposure (Mishra et al., 2013).

Therefore, the cross-resistance between AMP constituents of the immune system and therapeutic AMPs could be seen as a “two-way street.” Treatment with therapeutic AMPs could select strains that are cross-resistant to AMP constituents of the immune system, but previous exposure to the latter could select strains that are cross-resistant to therapeutic AMPs.

In a study with an experimental model of mealworm *Tenebrio molitor*, Dobson et al. (2014) showed that *S. aureus* with evolved iseganan-resistance increased survival in the host. This could support the “arming the enemy” hypothesis, because the long-lasting humoral immune response of this host is dependent on AMPs. However, the survival of the melittin-selected bacteria was not significant with respect to the ancestral bacteria, while the pexiganan-selected bacteria showed a trend toward high survival.

Most of the studies cited here showed that monotherapy with a therapeutic AMP could select resistant bacteria that potentially could cross-resist the action of AMP constituents of the innate immune system. However, there have also been studies where the selection of resistant bacteria via monotherapy with AMPs did not imply cross-resistance to AMPs of the immune system. Pränting et al. (2008) demonstrated that mutants of *Salmonella enterica* serovar Typhimurium in the gene *sbmA* that codes for the putative transport protein SbmA were resistant to PR-39, but the same mutants did not show cross-resistance to LL-37, CRAMP and rCRAMP. More recently, Narayanan et al. (2014) showed that *Escherichia coli* mutants in the *sbmA* gene were resistant to pyrrhocoricin. Nevertheless, any cross-resistance to LL-37 were observed.

Additionally it was demonstrated that combinatorial therapy of several AMPs or AMPs/antibiotics could circumvent the evolution of resistance and cross-resistance to innate immune system AMPs boosting it (Sakoulas et al., 2012; Dobson et al., 2013; Chernysh et al., 2015). It was observed that daptomycin/ampicillin combination was effective against an ampicillin- and vancomycin–resistant *Enterococcus faecium* isolate from a patient with endocarditis and bacteremia. Such strain was refractory to daptomycin/linezolid treatment. Furthermore, it was observed that ampicillin is able to enhance daptomycin bactericidal activity probably by reducing the increased positive net-charge surface commonly observed in resistant bacteria, which facilitate daptomycin binding. In the same study in presence of ampicillin, ampicillin- and vancomycin-resistant *Enterococcus faecium* were more susceptible to LL-37, hNP-1, and tPMPs (Sakoulas et al., 2012). Other study sheds that daptomycin/ampicillin combination was effective in killing daptomycin- non-susceptible enterococcal isolates with mutations in the *liaFSR* system. However, ampicillin could enhance the bactericidal activity of LL-37 against daptomycin- non-susceptible enterococcal isolates regardless of the presence of *liaFSR* mutation (Hindler et al., 2015).

## Conclusion

Bacterial resistance to AMPs has reached levels that are becoming a major concern, threatening the potential of AMPs as a therapeutic option. This phenomenon has become more dangerous because treatment with therapeutic AMPs could potentially induce cross-resistance toward AMP constituents of the innate immune system. Although there are few reports about this topic, the number has grown in recent years. Some studies have shown that monotherapy with therapeutic AMPs could select AMP-resistant bacteria that at the same time are cross-resistant to AMP constituents of the innate immune system and could persist in the host. This could compromise the host defense, based on the innate immune system. Therefore, it could be important to include tests of cross-resistance to host AMPs in the evaluation of new therapeutic AMPs. The findings also suggest that there may not be a precise correlation between *in vitro* cross-resistance data and *in vivo* data, highlighting also the importance of performing cross-resistance assays *in vivo*. Understanding the molecular basis of AMP cross-resistance is important for the development of more efficient therapeutic AMPs. Moreover, some studies revealed that use of AMPs in combination constrains the evolution of resistance to them and the combination of AMPs with other antimicrobials like β-lactams increase the likelihood of clinical success. Thus, the therapeutic based in AMPs could be address toward the design of therapeutic schemes based in the combinations of AMPs or AMPs/antibiotics for limited the evolution of resistance and cross-resistance, cover a broad spectrum of targets, potential the antimicrobial activity and boost the microbicidal activity of the AMPs-based innate immune system.

## Author Contributions

All authors listed, have made substantial, direct and intellectual contribution to the work, and approved it for publication.

## Conflict of Interest Statement

The authors declare that the research was conducted in the absence of any commercial or financial relationships that could be construed as a potential conflict of interest.

## Footnotes

1 The official term is “daptomycin non-susceptibility.”
